# Mechanical Properties and Strengthening Mechanism of Dredged Silty Clay Stabilized by Cement and Steel Slag

**DOI:** 10.3390/ma15113823

**Published:** 2022-05-27

**Authors:** Jian Shi, Shengnian Wang, Wenzhe Cao, Jun Su, Xingjin Zhang

**Affiliations:** 1 Nanjing Environment Group Co., Ltd., Nanjing 210014, China; shijian8928@126.com (J.S.); 18761876790@163.com (W.C.); 2 College of Transportation Science & Engineering, Nanjing Tech University, Nanjing 211816, China; 202061225084@njtech.edu.cn (J.S.); xingjinzhang@njtech.edu.cn (X.Z.)

**Keywords:** dredged soil stabilization, mechanical properties, strengthening mechanism, construction materials, performance improvement

## Abstract

The high moisture content and low strength of dredged soft soils result in significant difficulties in directly reutilizing them in engineering. Improving their mechanical properties effectively and achieving re-utilization with the maximum benefit in engineering is the key to disposing of dredged soils with high moisture content. This study investigated the influences of cement and steel slag ratio, moisture content, the maximum particle size of steel slag, and curing age on the compressive strength of dredged silty clay in a plastic flow state. The performance improvement of dredged silty clay stabilized with cement and steel slag was discussed by comparing to related previous studies. The strengthening mechanism of dredged soils stabilized with cement and steel slag was explored by microstructural observation. The results show that when the ratio of cement to steel slag was 9:6; namely, using steel slag to replace 40% of cement, the strength properties of dredged silty clay stabilized by cement and steel slag could ensure the minimum requirements of the project greater then 100 kPa, and their economics could achieve the best results. The finer the particle size of steel slag was, the better the stabilization effect was. The compressive strength of dredged silty clay stabilized by cement and steel slag with particle sizes of less than 0.075 mm was 1.06 times, 1.10 times, and 1.16 times that of 0.25 mm, 1 mm, and 2 mm and increased linearly over curing ages earlier than 28 days. The compressive strength of dredged silty clay stabilized by cement and steel slag cured for 28 days was 2.44 times, 1.59 times, and 1.36 times that of 3, 7, and 14 days, respectively. The evolution of microstructural characteristics showed that the internal pore sizes of dredged soil decreased the structural compactness increased significantly due to the formation of more calcium silicate hydrate and other agglomerated flocculent gel materials from the further reaction between steel slag and cement hydration products. The results of this study can provide technological parameters for the re-utilization of dredged soil stabilized with cement and steel slag.

## 1. Introduction

Dredged soils are sedimentary wastes containing organic matter excavated in waterway and port-dredging projects, primarily composed of chalky sand and fine grains with poor grading. They are commonly classified as typical super-soft soil in engineering due to high moisture content, insufficient bearing capacity, low shear strength, and poor permeability [1]. The dredged soils always contain high organic matter content, high limit moisture content, low specific gravity, slow consolidation and drainage, and difficulty in controlling their settlement and deformation. Thus, they are also regarded as problematic soils by the scientific and engineering communities [2]. Preliminary statistics illustrated that tens of billions of cubic meters of dredged soil were produced worldwide every year [3,4,5], among which billions of cubic meters were generated from the major rivers and commercial ports in China in 2021. How to effectively dispose of dredged soils and achieve resource utilization has been a hot issue, attracting scholars at home and abroad to pay close attention to due to its urgently need to be solved.

The key to the re-utilization of dredged soils is efficient and rapid stabilization of them. At present, the main treatment methods for soft soils with high liquid limits in engineering practices include stockpile drying, physical dewatering, and inorganic improvement. Stockpile drying is to set up a landfill for a long time or permanent stockpiling, in which the dredged soils are dried naturally and reused nearby at a later stage. Ding et al. [6] pointed out that three stages, corresponding to convective descent, dynamic collapse, and passive diffusion, were observed during settlement. This treatment method has simple operation and low cost and is widely used in areas where land resources are not in short supply. However, it is also accompanied by many problems, including longer drainage consolidation cycles, land occupation, transportation difficulties, secondary pollution, and high requirements for sediment pollution control [7]. Physical dewatering combines chemical precipitation and filter-press dewatering to reduce the moisture content of dredged soil to below 50% and then resource utilization after drying [8]. However, existing treatment technology and equipment capacity significantly limit the dewatering efficiency. Therefore, the more acceptable improvement for engineering practices is stabilizing dredged soils with inorganic binders, such as cement, lime, fly ash, etc. Jan and Mir [9] pointed out that the dredged soil stabilized with 8% lime could achieve the best mechanical properties and optimal compactness. Cheng et al. [10] summarized the empirical formulas of water/cement ratio, moisture content and curing age for cement-stabilized dredged soil. However, although large dosages of lime and fly ash can improve the physical state of the soil with relatively high moisture content, their strength improvement is slow. Similarly, although cement can improve the mechanical performance of soft soils with high moisture content to meet the specific engineering requirements, the cost of soil stabilization is the most significant pain point. Moreover, cement production consumes much energy, accompanying a high carbon emission, and the excessive utilization of cement is not friendly to the resource and eco-environment [11]. Therefore, engineering researchers have started to turn their attention to complex stabilization agents to reduce the consumption of cement and the cost of the project [12,13].

The basic idea of the complex stabilization method is to maximize the use of silica-aluminate-containing materials to replace cement without reducing the strength improvement effect of the soil stabilized with the same cement content, thereby achieving the satisfaction of engineering requirements and cost savings [14,15,16]. For example, cement has been often used for soil improvement by mixing fly ash and slag in road and water conservancy works. However, there is minimal research regarding the stabilization of soft soils with high moisture content. Kim et al. [17] and Liu et al. [18] indicated that the compressive strength of cement- and steel-slag-stabilized dredged soil had a negative correlation with the moisture content of the soil; Gençel et al. [19] and Cikmit et al. [20] found that the strength development of dredged soils stabilized with the utilization of steel slag as coarse and fine aggregates would be significantly affected by the size effect of steel slag. Lang et al. [21] reported that the compressive strength of dredged sludge increased with cement content, while the presence of humic acid severely hindered their strength development. They also addressed that the early strength of dredged soils could be effectively improved due to the rapid hydration of cement, while their later strength would be further enhanced as the reaction of steel slag with the products of cement hydration [22]. Peng and Chen [23] pointed out that the mechanical properties of MgO: GGBFS (ground granulated blast furnace slag) with 1:1 curing agent were best when 20% was incorporated into the dredged soil. He et al. [24] used soda residue (SR), carbide slag (CS), and ground granulated blast furnace slag (GGBFS) to stabilize grudged soil at high moisture content, indicating that the increase in GGBFS dosage can increase the unconfined compressive strength, and the optimum dosages of SR and CS were 35% and 6%, respectively. Thomas and Rangaswamy [25] investigated the strengthening of nano-silica on cement-treated soft clay via a series of strength and microstructural tests, and they proved a positive effect of nano-silica on the strength improvement of cement clay. Hence, a better understanding of the mechanical performance evolution of dredged soils stabilized with complex stabilization agents is the key to guiding their engineering application.

The utilization of steel slag could improve the physical state of the dredged silty clay and promote the hydration of cement, thereby accelerating the stabilization of dredged silty clay and saving cement. Therefore, this study prepared the complex stabilization agent with cement and steel slag for the stabilization of dredged silty clay. A series of indoor compression tests for dredged silty clay in a plastic flow state was conducted with different cement and steel slag ratios, moisture contents, particle sizes of steel slag, and curing ages. The optimal replacement ratio of steel slag to cement and the influence of these above factors on their compressive strength were investigated. The performance improvement of dredged silty clay stabilized with cement and steel slag was discussed by comparing to related previous studies. Furthermore, the strengthening mechanism of dredged silty clay stabilized by cement and steel slag at different curing ages was explored by scanning electron microscope (SEM).

## 2. Materials and Methods

### 2.1. Experimental Materials

(1) Dredged soil

The dredged soil used in the test was silty clay collected from the Class III waterway at the Zhejiang section of the Beijing-Hangzhou Canal. The sampling depth of this dredged soil was 4–6 m below the bottom of the canal. It was mainly yellowish-brown, fine granular particles and without organic and other impurities. The particle size composition of this dredged soil in <0.075 mm, 0.075–0.1 mm, 0.1–0.25 mm, 0.25–0.5 mm, 0.5–1 mm, and 1–2 mm accounted for 34.96%, 17.22%, 39.66%, 2.04%, 2.16%, and 3.96%, respectively. The natural moisture content was 23.6%. The specific gravity was 2.69. The liquid limit was 40.18%, the plastic limit was 24.27%, and the plastic index was 15.91.

(2) Cement

The cement used in this study was P.O.42.5 ordinary Portland cement with a fineness of 160 mesh, and its chemical composition is shown in Table 1.

(3) Steel slag

The steel slag used in the study was darker black particles with extreme heterogeneity in size. The chemical composition and content are shown in Table 2.

### 2.2. Experimental Scheme

**Figure 1:**
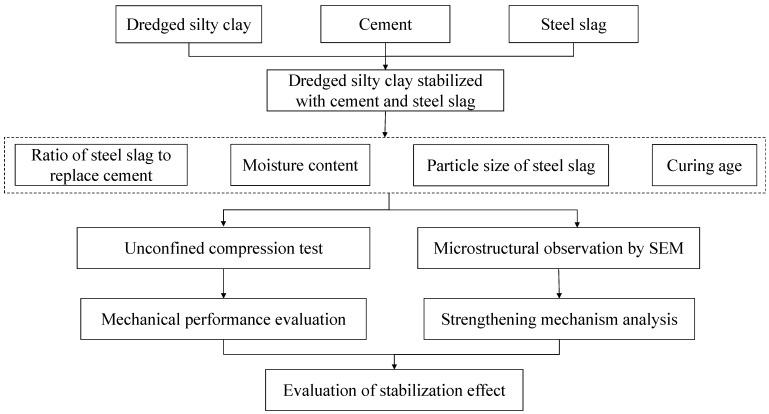
Systematic experimental technique route for this study.

The experimental study included three parts: (1) to explore the optimal ratio of steel slag to replace cement through compression tests on dredged soils with different moisture content; (2) to investigate the influence of particle size of steel slag and curing age on the strength properties of dredged soils stabilized by cement and steel slag; (3) to figure out the strengthening mechanism of dredged soils stabilized by cement and steel slag. Figure 1 presented a systematic experimental technique route for this study. The detailed experimental scheme is as follows:

(1) Tests for the optimal ratio of steel slag to replace cement

Many previous engineering practices showed that the dosage of cement used in soil stabilization was generally lying between 7% and15%. Considering significant differences in moisture content, unconfined compression tests on dredged soils with 1.0, 1.1, 1.15, 1.25, and 1.5 times the liquid limit were implemented with the same content (15%) of stabilization agent but different mixing ratios of cement and steel slag, including 12:3, 11:4, 10:5, and 9:6. The optimal ratio of steel slag to replace cement would be determined by taking 100 kPa as the reference standard. When testing, an universal testing machine was used to perform axial compression at a fixed rate of 1 mm/min until the specimen was destroyed.

(2) Tests for the law of strength improvement

Unconfined compression tests on dredged soils stabilized with 15% of cement and steel slag by following the optimal ratio of steel slag to cement were conducted first with 1.0, 1.1, 1.15, 1.25, and 1.5 times the liquid limit to understand the influence of moisture content on the strength improvement of untreated dredged soils, in which the maximum particle size of steel slag was 2 mm. Then, unconfined compression tests on dredged soils stabilized with 15% of cement and steel slag by following the optimal ratio of steel slag to cement were conducted, with the maximum particle size of steel slag as 0.075 mm, 0.25 mm, 1 mm, and 2 mm to study the strength improvement affected by the activity of steel slag particles in different sizes, in which the moisture content was constant as 1.25 times the liquid limit. Finally, unconfined compression tests on dredged soils stabilized with 15% of cement and steel slag by following the optimal ratio of steel slag to cement were conducted after different curing ages, including 3, 7, 14, and 28 days, to investigate the strength development of dredged soils stabilized with cement and steel slag, in which the moisture content was 1.25 times the liquid limit, and the maximum particle size of steel slag was 2 mm. Similarly, the universal testing machine would be used to perform axial compression at a constant rate of 1 mm/min until the specimen was destroyed.

(3) Tests for micro strengthening mechanism

Microstructural observation on dredged soils stabilized with 15% of cement and steel slag by following the optimal ratio of steel slag to cement was carried out by JSM-6510 scanning electron microscope (SEM) after 3, 7, 14, and 28 days, in which the moisture content was 1.25 times the liquid limit, and the maximum particle size of steel slag was 2 mm. The SEM images at the magnification of 500 times were used to analyze the structural compactness of the soil sample through the features of cutting surfaces, the contacts between large particles and small particles, and the pore size and distribution. The SEM images at the magnification of 5000 times were used to observe the characteristics of micro-particles and micro-pores and the formation of gels.

### 2.3. Specimen Preparation

(1) Compression Test

Cylindrical specimens preparing for unconfined compression tests were 39.1 mm in diameter and 80.0 mm in height. The dry dredged soil, cement, and steel slag were mixed well and then mixed with water to form a homogeneous slurry when beginning to sample. The water consumption was determined by the specified times of the liquid limit. The slurry of dredged soil, cement, and steel slag was poured into the mold with the same size of specimens several times and compacted by mechanical vibration. All specimens were stood in a curing box for 24 h at a temperature of 20 ± 3 °C and relative humidity of 95%. Then, they were demolded and cured under the same conditions until the curing age reached seven days.

(2) Microstructure Test

When the curing age of the prepared soil samples reached the specified curing age, they were cut, dried, polished, and flattened into small pieces of about 10 mm × 10 mm × 3 mm. Then these small pieces were sprayed with gold and vacuumed to prevent the absorption or scattering of the high-energy electron beam from hitting the air molecules during testing.

### 2.4. Experimental Apparatus

(1) Electronic universal testing machine

**Figure 2:**
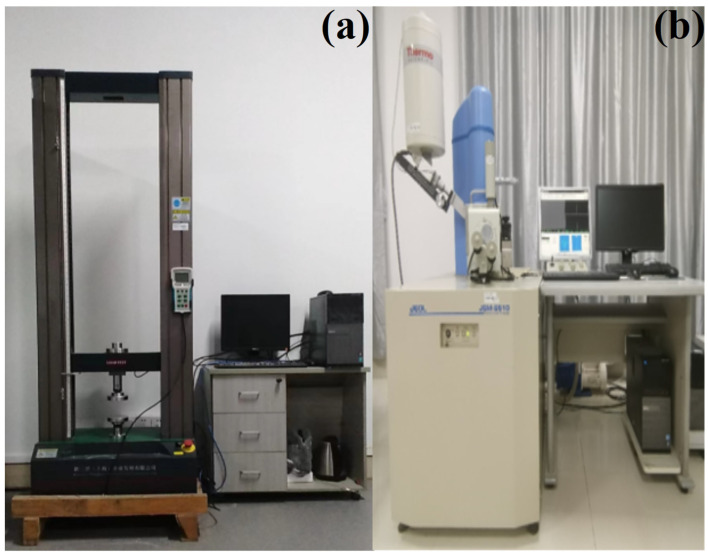
Experimental apparatus. (**a**) Universal testing machine, (**b**) Scanning Electron Microscope.

A microcomputer-controlled electronic universal testing machine was used to measure the compressive and tensile strength of dredged silty clay stabilized by cement and steel slag, as shown in Figure 2a. The designed maximum working frequency of this machine is 50.0 Hz. The ultimate testing force is 20 kN. The loading speed range is 0.001–500 mm/min with a control accuracy of ±1% (0.001~10 mm/min). The accuracy of deformation measurement is ±0.5%. The constant force and displacement ranges are 0.2–100%FS (FS is full scale). When beginning tests, the saturated specimen was placed on the loading platform, and the loading platform slowly rose to contact the upper plate first. Then, the stress and strain were initialized to be zero by the controlling software. Each sample was loaded at a 1 mm/min strain rate until appearing apparent deformation failure; then, manual loading was stopped.

(2) Scanning Electron Microscope

The Phenom Pro Desktop scanning electron microscope (SEM-JSM-6510), equipped with an Energy Dispersive Spectroscopy (EDS-NS7-7911), was adopted to observe the evolutions of microstructural characteristics and chemical composition content of silty sands after stabilization, as shown in Figure 2b. The accelerating voltage range of SEM-JSM-6510 is 5–30 kV. The minimum resolution of this device can be up to 3.0 nm. The structure characteristics of testing samples can be magnified 18–300,000 times. The imaging signal is collected by secondary electrons, backscattered electrons, and X-rays. When beginning to test, the sample is sprayed with a thin layer of gold and vacuumed to prevent the high-energy electron beam from colliding with air molecules and being absorbed or scattered during the test.

## 3. Results and Discussion

### 3.1. The Optimal Ratio of Steel Slag to Replace Cement for Dredged Soil Stabilization

**Figure 3:**
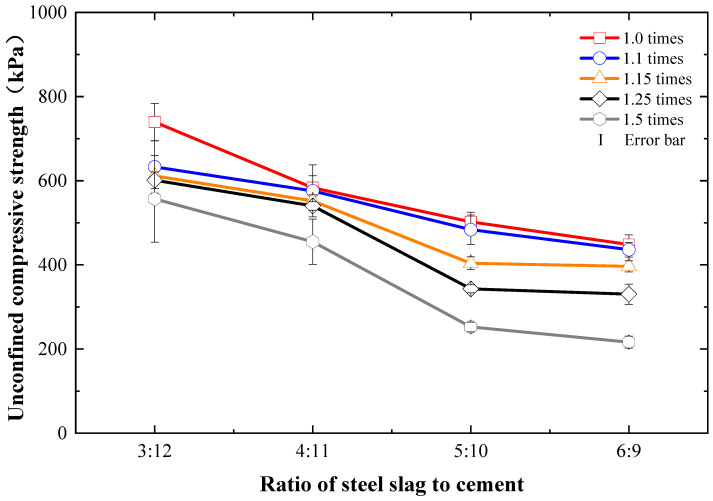
Compressive strength variations of cement- and steel-slag-stabilized dredged soils with different moisture contents and ratios of steel slag to replace cement.

Oh et al. [26] reported that a higher slag portion in the dredged soil stabilization could induce greater strength improvement. Gupta et al. [27] indicated that adding 8% cement + 8% slag in dredged soil could optimally satisfy the required acceptance norms of highway subgrade materials. Figure 3 shows the unconfined compressive strength of cement- and steel-slag-stabilized dredged soils with different moisture contents and ratios of steel slag to cement. It can be found that when the total dosage of cement and steel slag mixture was constant at 15%, the compressive strength of dredged soils stabilized by cement and steel slag decreased with the amount of steel slag overall regardless of the moisture content of the dredged soil. The more the relative amount of steel slag was, the more significant the reduction of compressive strength was. If the actual engineering demanded a compressive strength greater than 100 kPa as an early standard [28], the ratio of steel slag to cement as 5:10 or 6:9 could meet the engineering requirements on the compressive strength of the dredged soil even if the moisture content was 1.5 times the liquid limit. Considering that the compressive strength of the dredged soil stabilized by steel slag and cement with ratios of 5:10 and 6:9 was approximately equivalent, and the cement was a high energy consumption and production cost with a large amount of greenhouse gas emissions, the ratio of steel slag and cement as 6:9 should be an optimal economic ratio. Namely, the replacement ratio of steel slag to cement was 40%. This result was slightly different from that suggested by Lang et al. [21], in which the dredged soil of the Shanghai Minhang Channel was stabilized by 10% cement and 5% steel slag. The slight difference in their mechanical performance may be probably due to the differences in the fundamental physical properties of the dredged soil used in these two studies and the chemical composition and content of steel slag, which led to differences in the dosage of cement, thereby resulting in different strength improvement.

### 3.2. Influence of Moisture Content on the Compressive Strength of Dredged Soils Stabilized by Cement and Steel Slag

**Figure 4:**
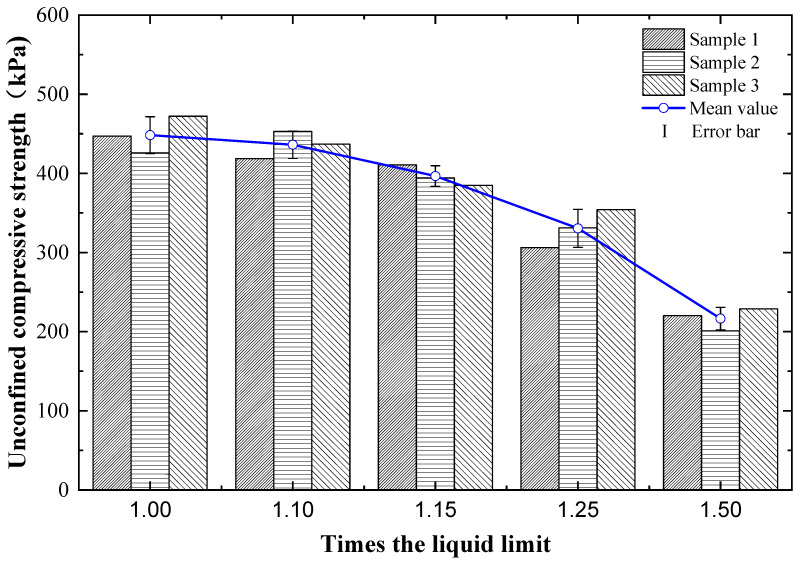
Compressive strength variations of cement- and steel-slag-stabilized dredged soils with different moisture contents.

Figure 4 presents the variation of the compressive strength of cement- and steel-slag-stabilized dredged soils with different moisture contents but following the same dosage of cement and steel slag. It can be found that the compressive strength of dredged soils stabilized by cement and steel slag decreased continuously as the moisture content increased from 1.0 times to 1.5 times their liquid limit. This variation may be due to the water resulting in soil softening with slow strength development. Previous studies reported by Kim et al. [17] and Liu et al. [18] indicated that the higher the moisture content was, the lower the compressive strength was. Results of this study also showed that when the moisture content was greater than 1.15 times the liquid limit, the compressive strength reduction of cement- and steel-slag-stabilized dredged soil became faster and faster, which implied that controlling the moisture content of dredged soil before stabilization was significant to enhance their mechanical performance. A more desirable stabilization effect would be achieved if the dredged soil was dewatered by an appropriate amount of water reducer and then stabilized by cement and steel slag.

### 3.3. Influence of the Particle Size of Steel Slag on the Compressive Strength of Dredged Soils Stabilized by Cement and Steel Slag

**Figure 5:**
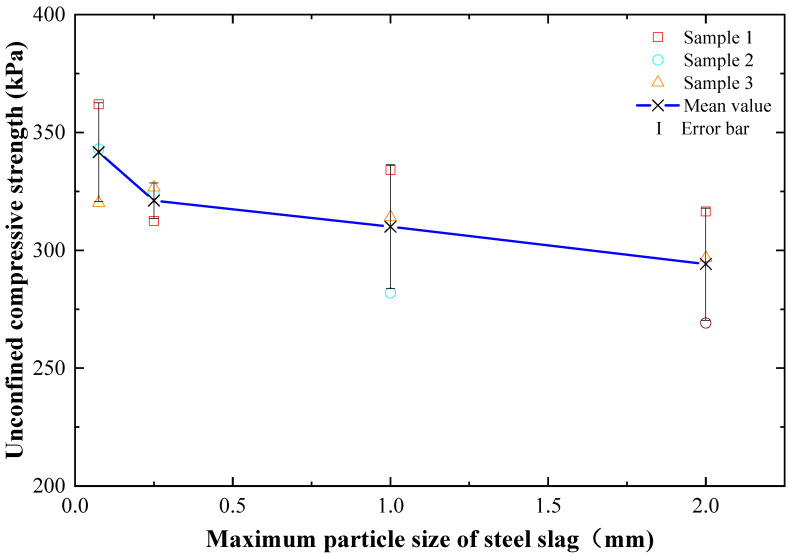
Compressive strength variations of cement- and steel-slag-stabilized dredged soils with different maximum particle sizes of steel slag.

Cikmit et al. [20] pointed out that the compressive strength was significantly affected by the maximum particle sizes of steel slag. The smaller the maximum particle sizes of steel slag, the better the mechanical performance that could be achieved. Figure 5 demonstrates the compressive strength changes of dredged soils stabilized by cement and steel slag with the different maximum particle sizes of steel slag, in which the ratio of steel slag to cement was 6:9. It can be observed that the compressive strength of dredged soils stabilized by cement and steel slag increased with the decrease of the maximum particle size of steel slag overall. This may be because the smaller the particle size of steel slag was, the larger the total specific surface area of steel slag was, the higher the activity of fine steel slag particles was, and the higher the pozzolanic reaction efficiency of steel slag particles with cement would be. The addition of steel slag promoted cement hydration, generating more gels such as calcium silicate hydrate (CSH) and calcium aluminate hydrate (CAH) during hardness so that the compressive strength of dredged soils stabilized by cement and steel slag was significantly improved.

### 3.4. Influence of Curing Age on the Compressive Strength of Dredged Soils Stabilized by Cement and Steel Slag

**Figure 6:**
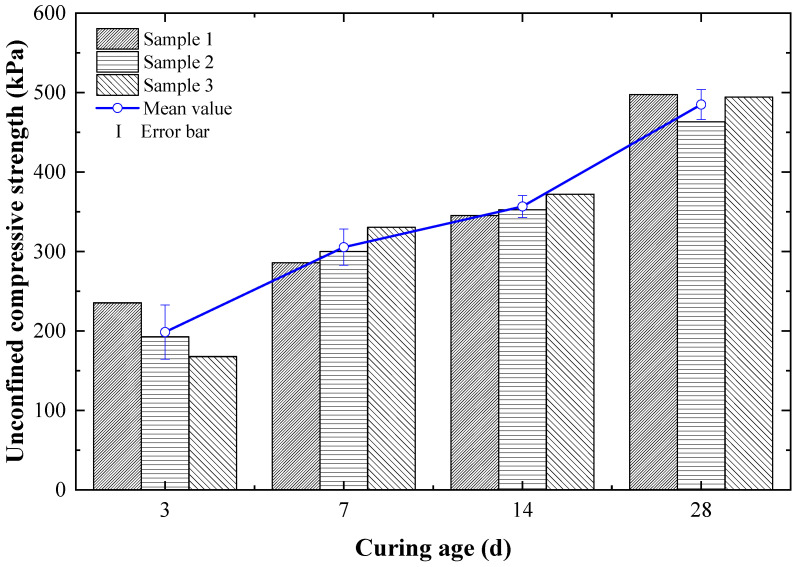
Compressive strength variations of cement- and steel-slag-stabilized dredged soils after different curing ages.

Figure 6 illustrates the variation of the compressive strength of cement- and steel-slag-stabilized dredged soils after different curing ages, in which the ratio of steel slag to cement was 6:9. It can be seen that the compressive strength of dredged soils stabilized by cement and steel slag increased over curing age. Cikmit et al. [29] and Lang et al. [30] achieved the same conclusion. When the curing age reached three days, the soil strength increased rapidly to 198.63 kPa; when the curing age was seven days, the strength exceeded 300 kPa, and when the curing age was 28 d, the strength reached 484.89 kPa. It can be also found that there was still no slowdown in the growth rate, which indicated that the addition of steel slag was similarly beneficial for the later compressive strength improvement of dredged soils stabilized by cement and steel slag.

### 3.5. Micro Strengthening Mechanism of Dredged Soil

**Figure 7:**
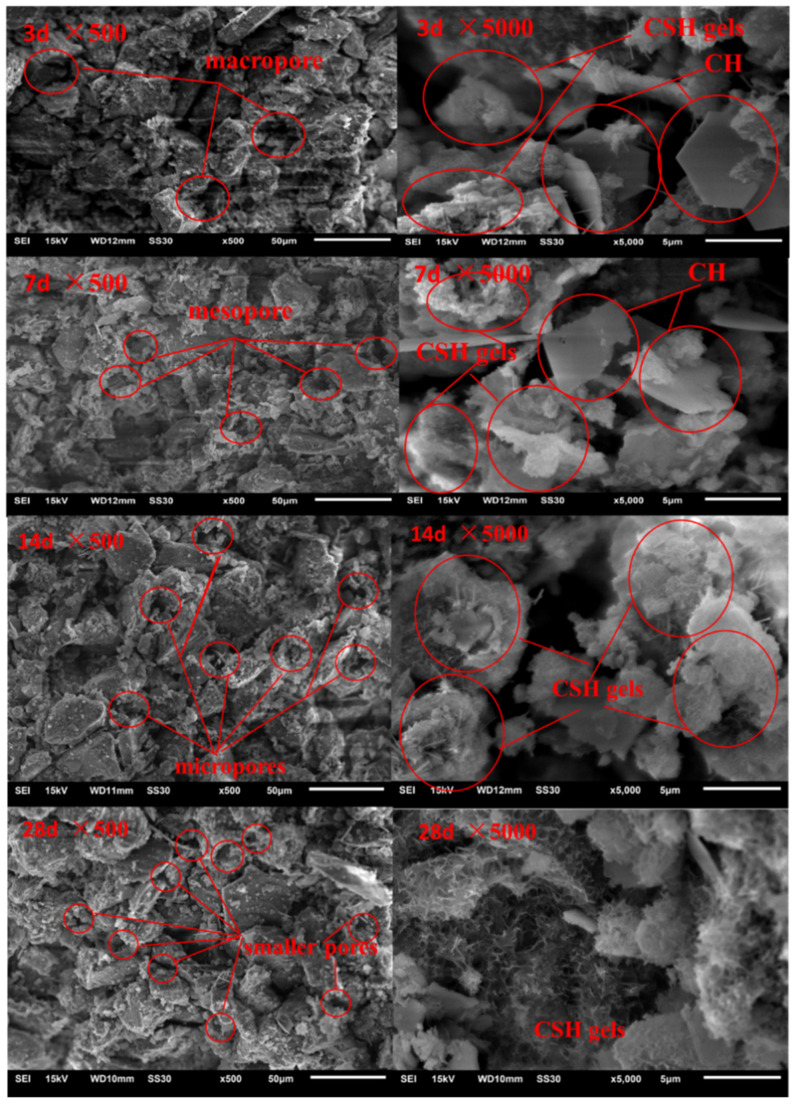
Microstructural characteristic evolutions of dredged soils stabilized by cement and steel slag at different curing ages.

Figure 7 shows the microstructure images of dredged soils stabilized by cement and steel slag at different curing ages. The microstructural characteristics of cement- and steel-slag-stabilized dredged soil at the magnitude of 500 times demonstrated that the pore size in the soil decreased with the development of curing age, and the agglomeration of soil particles became more and more significant. After curing for 28 days, most of the large pores had disappeared, and only relatively uniformly tiny pores remained. This fact indicated that the structural compactness of dredged soil had improved significantly. The microstructural characteristics of cement- and steel-slag-stabilized dredged soil at the magnitude of 5000 times presented that there were a large amount of microscopic, needle-like calcium silico-aluminate gels and hexagonal plate calcium hydroxide being formed on the surface of soil particles after curing for three days, which indicated that the hydration of cement was being carried out rapidly. When the curing age reached seven days, the needle-like calcium silico-aluminate gels extended and filled into large pores, making the volume of large pores shrink continuously and the amount of calcium hydroxide decrease, which might be caused by the further reaction of calcium hydroxide with steel slag particles that possessed a high volcanic ash activity to form more CSH and other gels, which can attach to the clay clusters and fill the pore spaces between clay particles, resulting in a denser sediment structure [31,32]. When the curing age reached 14 days, the calcium hydroxide produced by cement hydration disappeared, and the production of flocculent gels increased significantly, wrapped on the surface of soil particles, thereby shrinking the internal pores of dredged soils. When the curing age exceeded 28 days, the hydration and the pozzolanic reaction of the soil was completed, and the agglomerated flocculent cementitious material increased, wrapped the soil particles, and closed the pores, leaving only many smaller pores, which indicates that the cement steel slag can indeed serve the purpose of reducing the internal pores of dredged soils and improving their compressive properties. Lang et al. [22] also confirmed that the addition of steel slag powder can contribute to the formation of cementitious products in cement-stabilized dredged sludge, which can effectively improve its strength.

However, it should be noted that although this study could provide some reference for the stabilization of dredged soils with high moisture content, there are some limitations, such as the reaction mechanism of cement and steel slag and the representativeness of microstructural evolution [33,34]. The microstructural observation could give more information about the microstructural changes of cement- and steel-slag-stabilized dredged soils at different curing ages but was difficult to interpret how cement and steel slag react [22,35,36]. The aluminosilicate minerals in steel slag might react with the secondary product of cement hydration and also might copolymerize in the alkaline environment formed by the cement hydration to generate geopolymer. These two reactions were totally different. Similarly, the microstructural images could reflect the characteristics of observation points, but they could not represent the attribution of the whole sample, even all samples. Therefore, a more in-depth investigation is still needed for further study.

## 4. Conclusions

The re-utilization of dredged soil with different moisture contents is crucial to improving engineering economic benefits and the risk avoidance of environmental pollution. This study explored the mechanical performance and micro-strengthening mechanism of dredged soils stabilized by cement and steel slag through a series of unconfined compression tests. The outcomes show that the compressive strength of dredged soils stabilized by cement and steel slag decreased with the relative amount of steel slag regardless of the moisture content overall. The optimal economic ratio of steel slag to cement was 6:9, namely using steel slag to replace 40% of cement if the dosage of cement and steel slag used for soil stabilization was constant. A more desirable stabilization effect would be achieved if dredged soils were dewatered by an appropriate amount of water reducer and then stabilized with cement and steel slag. The steel slag could promote the hydration of cement. The smaller the particle size of steel slag was, the higher the pozzolanic reaction efficiency of steel slag particles with cement would be, and the more significant the strength improvement of dredged soil stabilized by cement and steel slag was. The compressive strength of dredged soil stabilized by cement and steel slag increased over curing age. The early strength of dredged soils could be effectively improved due to the rapid hydration of cement, while their later strength would be further strengthened by the reaction of steel slag with the products of cement hydration. The evolution of microstructural characteristics proved the contribution mode of steel slag to the strength improvement of dredged soil with the development of curing age. The results of this study can provide a reference for the application of dredged soils stabilized by cement and steel slag. However, further research on the reaction mechanism of cement and steel slag is still encouraged.

## Figures and Tables

**Table 1:** Chemical composition of cement used in this study.

Component	CaO	SiO_2_	Al_2_O_3_	Fe_2_O_3_	SO_2_	MgO	F-CaO	Loss on Ignition
Mass ratio (%)	57.22	23.19	8.61	4.08	1.93	1.04	1.37	2.56

**Table 2:** Chemical composition of steel slag used in this study.

Component	CaO	SiO_2_	MgO	Al_2_O_3_	Fe_2_O_3_
Mass ratio (%)	59.23	29.02	3.89	1.40	6.46

## Data Availability

The raw data supporting the conclusions of this article will be made available by the authors without undue reservation.

## References

[1] Mir B.A., Wani K.M.N.S. (2021). Mechanical Behavior of Boulder Crusher Dust (BCD)-stabilized Dredged Doil. Problematic Soils and Geoenvironmental Concerns.

[2] Develioglu I., Pulat H.F. (2019). Compressibility Behaviour of Natural and Stabilized Dredged Soils in Different Organic Matter Contents. Constr. Build. Mater..

[3] Howard I.L., Vahedifard F., Williams J.M., Timpson C. (2018). Geotextile Tubes and Beneficial Reuse of Dredged Soil: Applications Near Ports and Harbours. Proc. Inst. Civ. Eng. -Ground Improv..

[4] Kalianan S., Chan C. (2017). 1-D Compressibility Parameters of Lightly Solidified Dredged Marine Soil (DMS) Using Cement, GGBS and Coarse Sand. Int. J. GEOMATE.

[5] Shahri Z., Chan C. (2015). On the Characterization of Dredged Marine Soils From Malaysian Waters: Physical Properties. Environ. Pollut..

[6] Ding W., Lu C., Xie Q., Luo X., Zhang G. (2022). Understanding the Settling Processes of Dredged Sediment Disposed in Open Waters through Experimental Tests and Numerical Simulations. J. Mar. Sci. Eng..

[7] Smith B.T., Howard I.L., Vahedifard F. (2017). Lightly Cemented Dredged Sediments for Sustainable Reuse. Environ. Geotech..

[8] Qi Y., Thapa K.B., Hoadley A.F.A. (2011). Application of Filtration Aids for Improving Sludge Dewatering Properties—A Review. Chem. Eng. J..

[9] Jan O.Q., Mir B.A. (2018). Strength and Micro Structural Behavior of Lime Stabilized Dredged Soil. International Congress and Exhibition “Sustainable Civil Infrastructures: Innovative Infrastructure Geotechnology”.

[10] Cheng X., Chen Y., Chen G., Li B. (2021). Characterization and Prediction for the Strength Development of Cement Stabilized Dredged Sediment. Mar. Georesour. Geotec..

[11] Toniolo N., Boccaccini A.R. (2017). Fly Ash-Based Geopolymers Containing Added Silicate Waste. A Review. Ceram. Int..

[12] Dungca J.R., Ang K.D., Isaac A.M.L., Joven J.J.R., Sollano M.B.T. (2019). Use of Dry Mixing Method in Fly Ash Based Geopolymer as a Stabilizer for Dredged Soil. Int. J. GEOMATE.

[13] Kirgiz M.S. (2016). Advancements in Mechanical and Physical Properties for Marble Powder–Cement Composites Strengthened by Nanostructured Graphite Particles. Mech. Mater..

[14] Bazne M.O., Howard I.L., Vahedifard F. (2017). Stabilized Very High–Moisture Dredged Soil: Relative Behavior of Portland-Limestone Cement and Ordinary Portland Cement. J. Mater. Civ. Eng..

[15] Bazne M.O., Vahedifard F., Howard I.L. (2018). Effects of Light Cement Stabilization On Properties of Fine Grained Dredged Soils. Geotech. Test. J..

[16] Xu Q., Ji T., Gao S., Yang Z., Wu N. (2019). Characteristics and Applications of Sugar Cane Bagasse Ash Waste in Cementitious Materials. Materials.

[17] Kim Y.T., Ahn J., Han W.J., Gabr M.A. (2010). Experimental Evaluation of Strength Characteristics of Stabilized Dredged Soil. J. Mater. Civ. Eng..

[18] Liu Q., Li H., Sun X., Cong S., Zhu J., Deng Y. (2020). Application of Steel Slag Composite in In-Situ Solidification of Shallow Soft Soil. J. Disaster Prev. Mitig. Eng..

[19] Gençel O., Karadag O., Oren O.H., Bilir T. (2021). Steel Slag and its Applications in Cement and Concrete Technology: A Review. Constr. Build. Mater..

[20] Cikmit A.A., Tsuchida T., Kang G., Hashimoto R., Honda H. (2019). Particle-Size Effect of Basic Oxygen Furnace Steel Slag in Stabilization of Dredged Marine Clay. Soils Found..

[21] Lang L., Liu N., Chen B. (2020). Strength Development of Solidified Dredged Sludge Containing Humic Acid with Cement, Lime and nano-SiO2. Constr. Build. Mater..

[22] Lang L., Song C., Xue L., Chen B. (2020). Effectiveness of Waste Steel Slag Powder On the Strength Development and Associated Micro-Mechanisms of Cement-Stabilized Dredged Sludge. Constr. Build. Mater..

[23] Peng L., Chen B. (2021). Mechanical Behavior, Durability, Thermal Performances and Microstructure of GGBFS–Modified MPC Solidified Dredged Sludge. Constr. Build. Mater..

[24] He J., Shi X., Li Z., Zhang L., Feng X., Zhou L. (2020). Strength Properties of Dredged Soil at High Water Content Treated with Soda Residue, Carbide Slag, and Ground Granulated Blast Furnace Slag. Constr. Build. Mater..

[25] Thomas G., Rangaswamy K. (2020). Strengthening of Cement Blended Soft Clay with Nano-Silica Particles. Geomech. Eng.

[26] Oh M., Yoon L.G., Yoon Y.W. (2016). Evaluation On the Compressive Strength of Dredged Soil-Steel Slag. Jpn. Geotech. Soc. Spec. Publ..

[27] Gupta A., Biswas S., Arora V.K. (2020). Ranking of Stabilizers to Stabilize/Solidify Dredged Soil as Highway Construction Material. Mater. Today Proc..

[28] Sato T. (2020). Effect of Soil Organic Matters in Dredged Soils to Utilization of their Mixtures Made with a Steel Slag. Materials.

[29] Hossain M.B., Barman Z., Dey M. (2021). Properties of Locally Available River Dredged Soil Stabilized with Cement. Progress. Agric..

[30] Lang L., Chen B., Chen B. (2021). Strength Evolutions of Varying Water Content-Dredged Sludge Stabilized with Alkali-Activated Ground Granulated Blast-Furnace Slag. Constr. Build. Mater..

[31] Yoobanpot N., Jamsawang P., Simarat P., Jongpradist P., Likitlersuang S. (2020). Sustainable Reuse of Dredged Sediments as Pavement Materials by Cement and Fly Ash Stabilization. J. Soil. Sediment..

[32] Toda K., Sato H., Weerakoon N., Otake T., Nishimura S., Sato T. (2018). Key Factors Affecting Strength Development of Steel Slag-Dredged Soil Mixtures. Minerals.

[33] Tiwari N., Satyam N., Patva J. (2020). Engineering Characteristics and Performance of Polypropylene Fibre and Silica Fume Treated Expansive Soil Subgrade. Int. J. Geosynth. Ground Eng..

[34] Afolayan O.D., Olofinade O.M., Akinwumi I.I. (2019). Use of some Agricultural Wastes to Modify the Engineering Properties of Subgrade Soils: A Review. J. Phys. Conf. Ser..

[35] Yu L., Xia J., Xia Z., Chen M., Wang J., Zhang Y. (2022). Study On the Mechanical Behavior and Micro-Mechanism of Concrete with Coal Gangue Fine and Coarse Aggregate. Constr. Build. Mater..

[36] Zhao K., Wang Q.Z., Zhuang H.Y., Li Z.Y., Chen G.X. (2022). A Fully Coupled Flow Deformation Model for Seismic Site Response Analyses of Liquefiable Marine Sediments. Ocean Eng..

